# Chicago Medical Society

**Published:** 1887-04

**Authors:** 


CHICAGO MEDICAL SOCIETY.

*Stated Meeting, February 21st, 1887.*—THE PRESIDENT, E. J. DOERING, M. D., IN THE CHAIR.

The discussion of PROFESSOR J. A. ROBISON's paper on

CLIMATIC TREATMENT OF DISEASE

being in order,

PROFESSOR JOSEPH P. ROSS said he was called upon to open the discussion of the treatment by climate of consumption, one of the most frequent and fatal diseases that afflict our race. Statisticians tell us that more than three million persons die annually of this disease. Dr. G. B. Wood, in his "Practice of Medicine," states that north of the tropics one-sixth of all deaths are caused by this disease. Upon consulting Zeimssen he found that the author of the article on consumption makes the statement that two-sevenths of all the deaths in the world are caused by pulmonary tuberculosis. Since this disease is so general, and since treatment by medicine does not accomplish as much as hygiene, which includes climatic treatment, it is very important that we discuss the subject.

In the excellent paper read by Dr. Robison at the last meeting, he quoted the opinions of three leading medical men with reference to the kind of climate which is appropriate for the treatment of consumption, and Professor Ross thought their views with reference to the appropriate climate, which were adopted in the paper, are correct. From his study and observation of the effects of various climates upon tubercular patients, he believed that, although one cannot say any particular climate is suitable for every case, one can say that for cases in general the air should be pure and dry; should be neither too hot nor cold; should not be variable, and should be somewhat rarified; for instance, such air as would exist at an altitude of from 1,000 to 3,000 feet above the sea-level; and lastly, the air should be invigorating, because of its containing an additional element, probably ozone. It may be that this invigorating quality is due to an electrical condition of the air, but it is more likely due to ozone. He waived the discussion of each of these elements appropriate to a good atmosphere, but spoke more particularly of different localities that are known as health-resorts, and stated what facts his experience and study had taught him concerning these localities.

At one time the eyes of the medical profession were turned to Colorado as offering the best promises for a good climate for tubercular patients. It was thought that in Colorado we have a climate where there is pure, bracing air, with an altitude high enough to secure rarifaction of the air sufficient to invigorate the respiratory functions. He had observed this fact: that while the patients seem to improve in their general health, the exudate increases rapidly, and if softening occurs it takes place also very rapidly. This is probably due to the high altitude. He had also observed that in the high altitudes patients who have a tendency toward hemorrhages are apt to be unfavorably affected in this way. He considers that in the later stages of tuberculosis, the high mountains of Colorado and New Mexico are objectionable. For this reason only carefully selected cases are advised to go to Denver, Colorado Springs, Pueblo, Raton Pass, Las Vegas, Santa Fè and Albuquerque.

The advantages and the disadvantages of San Antonio, Texas, as a health-resort, were considered. During the winter some seem to improve, but a large number of them complain that the sudden cold winds from the north, or northers, as they are called, cause severe attacks of bronchitis or pneumonia. In the spring or summer there is a great deal of malaria. But barring the northers and malaria, a great many cases do very well there, and during the months of December and January it seems to be a very good climate for consumptives. But during the summer months the heat has too depressing an influence on the patients.

All things considered, he believes the best climate and opportunities for treating this disease are found in California. With reference to Southern California, probably for two months of the year it would be desirable to send patients down to Los Angeles, Riverside or San Diego; but taking that climate all the year round it is not the place, because the air does not contain the qualities that should make it essentially invigorating; it is not light enough, and seems to lack ozone. Patients affected with ordinary catarrh will do well there, but consumptive patients do not improve in Southern California all the year round. If one could live in the southern climate for two months in the year, and then go up into the Sacramento valley, where one can find almost any altitude, it would probably be the best climate for cases of consumption in general on the continent. One objection to sending patients there from Chicago is the distance.

Study of the Gulf coast and Florida led him to the conclusion that the climate of Florida is a failure for the treatment of consumption, for this reason: patients go there having a cough, being debilitated by disease, and the air is balmy, and they say, what a beautiful air! They do not cough so much, but they sit around and take no exercise, for there is nothing to cause them to take active exercise. There is something lacking in the air: it does not brace one up; he had never seen a patient there that improved the least from day to day. The atmosphere is warm and moist, and very depressing, while malaria abounds.

With reference to the regions mentioned in Professor Robison's paper—viz.: the mountains of Tennessee, Georgia and North Carolina—he said he fully agreed with the remarks made by the author, and thought the climate of these regions is suitable for the treatment of consumption. It is well to consider the favorable features of this mountainous region of Tennessee, Georgia and North Carolina, which is called the Switzerland of America, and to contrast the climate of that region with that of Chicago at the time of the year when our climate is bad. It is from January until June that our climate is bad—the latter part of winter and spring. Now, it is a fact that at an altitude of 6,000 feet above the sea level, as at Denver and Colorado Springs, the air is too cold; and when we have bad weather here, one will find bad weather in Colorado, extending from January until near June. During these months when our climate is bad, we must consider the question of sending patients away from Chicago. In Great Britain and the north of Europe they consider that the bad time of the year is from January to June, and they send patients down where the Alps dip into the Mediterrean Sea and where they find the climate mild—about the same conditions of climate that we have in some of our Southern States. All the rest of the year we have a good climate; in fall, summer and early winter, the Chicago climate is not bad.

In conclusion he said his reasons for recommending the South as a place to which to send consumptives are:

1. When the climate in Chicago is unsuited for consumptive patients, the climate is mild and favorable in the Southern States.

2. The nearness of Chattanooga, Marietta and Asheville to Chicago and the North, so that the trip can be made in a few hours, recommends it.

3. The results of a limited experience have convinced him that his patients do as well, if not better, at these points than at any other.

DR. J. J. M. ANGEAR said: He endorsed nearly all that Professor Ross has said, and most of the paper of Professor Robison. A few points in the paper he thought might be amplified and supplemented profitably. The author speaks of home comforts, pleasing scenery and congenial society, and these three things demand a good deal of attention. We are all aware of the fact that there is some truth in the old saying: "Laugh and get fat." It is an impossibility for any one to do well who is constantly under a cloud; it has a depressing influence upon the mind, it matters not how good the climate. The physician advises his patient to leave home for a more salubrious climate, and under the pressure of circumstances he takes his departure. It is probable that there would be more cheerfulness at home than away among strangers, no matter how desirable these places may seem. These things must be taken into consideration. He has believed for a number of years that the benefit of our watering places was not so much from the water, but because the patient leaves home, leaves business cares, and has a gay, good time—sadness of heart has melted away. That patient can "laugh and get fat." There is something here for us to study: the tranquility and peace of mind, so that nutrition can go on undisturbed.

He said the idea is advanced in the paper that in good environments bacteria cannot thrive. If they cannot thrive they are not able to do their mischief. There may be something in this which we do not understand. We do not believe that laughter will ever get rid of a tapeworm; we do not believe that cheerful society and good music will cast out or put a check upon the ravages of trichinæ; but we are compelled by force of circumstances to acknowledge the truthfulness of the assertion that good environments will check, if not destroy, the influence of microbes, and if we are convinced that microbes are really the cause of tuberculosis, then we want to place the patient in such an environment as will check, if not destroy their ravages. It may be wise for us to select pure air, the right temperature, and sunshine; but unless the patient can carry contentment with him, sunshine in his soul, it would be better for him to stay at home; and unless he has abundant means, so that he may not feel pinched and can enter into the gaieties of the place, he had better stay at home.

DR. J. G. KIERNAN said: The point raised by Dr. Angear, as to the mental condition having an influence on the progress of the disease, is valid as to principle, but he has ignored the mental peculiarities of the consumptive patient. Hope has been known as a characteristic for a long time, but the suspicious element is less known and as frequently characteristic. In removing a patient who has tuberculosis from the surroundings of his home we are doing the best possible for that patient, from a moral standpoint.

PROFESSOR R. G. BOGUE said: Some years ago it was his fortune to spend a few years in the South, the greater portion of them in the mountain regions of Tennessee, Northern Alabama and Northern Georgia. All of one winter, about six months, he spent in Chattanooga; and he could bear testimony to the wholesomeness of the entire region for the greater part of the year, and in the mountain regions for all of the year; in the valleys there are about two months in the summer season when malaria is quite prevalent, but up in the mountains it is eminently a healthy country. The air is pure, the water is good, the scenery is magnificent; there is everything to entertain and amuse one, so far as scenery is concerned. The region of Chattanooga is peculiar and delightful from the variety of the mountain and valley scenery. The entire year, or very nearly so, it is practicable for people to be out of doors. During the latter part of December, all of January, and the early part of February, the temperature is subject to considerable variation; it is reasonably cold and there are some storms, but after the middle of February, with the exception of now and then a short storm, the climate is delightful, extending thus through the whole summer. The altitude in the mountain region is such as to render the summer not oppressive. He believed that for the colder and stormy portion of the winter the region south of Atlanta might be better for consumptive patients than north of Atlanta. Macon, and that part of Georgia fifty miles north of the coast, and extending through Georgia and Alabama to the region of Mobile, are good localities. Quite early in the spring one can come northward. Coming north of that range of mountains into the valley this side of North Atlanta, to Marietta, one finds a delightful spring climate, with a great deal of sunshine, and a forest filled with bloom for two months in the spring; everything, as far as climate and scenery are concerned, to delight even a sick person. There is much to recommend the region of Chattanooga to invalids, and if they are not satisfied with Chattanooga or the vicinity, there are other points, Marietta, Atlanta, and north of the Tennessee river, the region of Huntsville, and north from that as far as Nashville; so that consumptive patients or invalids may be in a mild invigorating temperature, really all the year, in the space of a couple of hundred miles.

PROFESSOR WM. T. BELFIELD presented a

### PROSTATIC MYOMA—A SO-CALLED "MIDDLE LOBE" OF THE HYPERTROPHIED PROSTATE—REMOVED BY SUPRA-PUBIC PROSTATOTOMY.

The patient, a man 73 years old, had for several years experienced difficulty in urination, and for nearly a year had been practically dependent upon his catheter. There was found symmetrical enlargement of the prostate per rectum, dilatation and catarrh of the bladder, and an impediment at the bladder neck to the entrance of rigid instruments. Exploration of the bladder by supra-pubic incision revealed a solid prostatic outgrowth, or "middle lobe," as large as a hazel nut and of flattened pear-shape, springing by a short, narrow pedicle from the vesical orifice. The pedicle was twisted off with forceps and the growth removed. Recovery was uninterrupted, the fistula closing entirely on the seventeenth day. Patient has since urinated freely without a catheter, and can now almost completely empty the bladder; the cystitis has subsided.

PROFESSOR BELFIELD also presented specimens of

MUCOUS CASTS FROM A CASE OF MEMBRANOUS ENTERITIS.

The patient was a nervous, rather hysterical lady about 35 years of age, who for several years had at intervals suffered from severe intestinal colics, followed in a day or two by diarrhœa with the expulsion of these casts; for several days thereafter much soreness and tenderness of the abdomen was experienced. The casts appear under the microscope as amorphous pseudo membrane enclosing cast-off epithelial cells. They are said by DaCosta to give the chemical reactions of mucin. This patient improved materially under small doses of bichloride of mercury and pills of iron, arsenic and strychnine.

He also exhibited a

FOREIGN BODY FROM THE BLADDER; a roll of chewing-gum, two inches long, partially encrusted with urinary salts, which had been removed from the bladder of a young man by Professor T. W. Miller. The patient, suspecting he had a stricture, had explored his urethra with a well-masticated roll of chewing-gum stuck upon the end of a broom-straw. When the straw was withdrawn the gum had disappeared. Acute cystitis ensued. Attempts to detect and extract the gum with a small lithotrite having failed, perineal urethrotomy was performed and the gum extracted by the finger. The patient entirely recovered in two weeks.

He also presented a

FRAGMENT OF THE OCCIPITAL BONE

of irregular quadrilateral shape, three inches long and two and one-fourth inches broad, its longest edge serrated for articulation with the right parietal bone. The fragment was removed by him from a young man who had suffered a compound comminuted fracture of the skull, by a blow with a sharp iron instrument which had penetrated the brain to depth of over an inch. On admission to the Cook County Hospital, over a teaspoonful of brain substance was found among the hair; the scalp and dura mater were badly lacerated. The fragments of bone were removed, the wounds in the brain substance cleansed and drained, and the scalp wound sewed up. After the fourth day the temperature remained normal; the wound was entirely healed in a month, the dressing having been changed four times during that period. The scalp is depressed over the seat of fracture, and still pulsates with the brain.

He then also presented a specimen of

INTESTINE COVERED WITH MILIARY TUBERCLES, DEATH HAVING RESULTED FROM MILIARY MENINGITIS.

This specimen is a piece of intestine the peritoneal covering of which is full of miliary tubercles. The patient was a girl about twenty years old, who had always had excellent heath. Three sisters are still living, and neither they nor their parents, nor any of their relatives so far as known, have ever shown symptoms of tuberculosis. The present generation are examples of perfect health, and this girl was in many respects apparently the healthiest of all of them, Until five days before her death her health was as usual; then she complained of feeling ill one evening; her head ached; she vomited, without apparent cause; she lay down, drank a cup of tea and felt all right. The same thing happened the next day, and she complained of extreme headache. On the third day, with the exception of severe headache and vomiting without cause, she maintaind her usual health. The evening before her death she was at a theater, and came home about 11 o'clock and went to bed with one of her sisters. During the night the sister was several times awakened by the extraordinary breathing of the patient, which was very hard and loud. She shook her, and the abnormal breathing ceased. In the morning it was found impossible to wake her; she was breathing very deeply and slowly, and could not be aroused. Physicians were summoned and all of them pronounced it a case of opium-poisoning. The symptoms as related to me were stertorous breathing, sometimes as slow as four or five a minute ,contracted pupils, a suggestion of strabismus, and rather warm surface. The pulse was remarkably good, about 80, similar to the pulse of a healthy person; the skin was not moist and clammy. The physicians instituted the usual measures for opium-poisoning; they gave mustard at once, and as soon as a tube could be procured pumped out the stomach. They injected minute doses of atropine hypodermically; the patient received about a fortieth of a grain in three injections. The breathing improved very much after these injections, coming up to twelve and fifteen per minute; the pulse also quickened; suddenly the pulse flickered and went out, and the breathing stopped.

On the following day, with the Assistant County Physician, he made a post-mortem examination, which included the head and abdominal cavity only. The brain was not hyperæmic, there was no venous congestion, the pupils were widely dilated; the membranes of the brain were adherent to the brain substance, and on stripping them off and examining them there were found numerous miliary tubercles. On opening the abdominal cavity the intestine bulged out, its surface thickly studded with miliary tubercles; there was extreme tuberculosis of the peritoneum, and the mesenteric glands were large and cheesy; there was a ruptured cyst of the right ovary, about as large as a fist. That the rupture was ante-mortem was evident from the fact that the inner surface of the cyst was intensely congested; there was a good deal of coagulated blood in its cavity. While it would be impossible to say that no opium had been taken, yet it seems probable, when we consider that the pulse was natural, that the surface was not cool and clammy, and that there was a tendency to strabismus, that her death resulted from the tuberculosis, perhaps rupture of the cyst. There was no morphine found anywhere around, and her general disposition and circumstances were such as to forbid the conception by her friends that she could have taken poison. The case seems to have been another instance of sudden coma incident to tubercular meningitis. It is of extreme interest as showing how insidious miliary tuberculosis of the serous membranes may be. The only abnormal feature known to her family was an unusual fullness and hardness of the abdomen, developed during the last year of her life.

